# Supplementary material for: ‘Care Under Pressure’: a realist review of interventions to tackle doctors’ mental ill-health and its impacts on the clinical workforce and patient care
Source: BMJ Open. 2018 Feb 2;8(2):e021273. doi: 10.1136/bmjopen-2017-021273 (PMC5829880; doi:10.1136/bmjopen-2017-021273)
Supplement: Author's manuscript [file bmjopen-2017-021273.draft_revisions.pdf]

BMJ Open

BMJ Open is committed to open peer review. As part of this commitment we make the peer review history of every article we publish publicly available.

When an article is published we post the peer reviewers' comments and the authors' responses online. We also post the versions of the paper that were used during peer review. These are the versions that the peer review comments apply to.

The versions of the paper that follow are the versions that were submitted during the peer review process. They are not the versions of record or the final published versions. They should not be cited or distributed as the published version of this manuscript.

BMJ Open is an open access journal and the full, final, typeset and author-corrected version of record of the manuscript is available on our site with no access controls, subscription charges or pay-per-view fees (http://bmjopen.bmj.com).

If you have any questions on BMJ Open's open peer review process please email editorial.bmjopen@bmj.com

BMJ Open

'Care Under Pressure': a realist review of interventions to tackle doctors' mental ill-health and its impacts on the clinical workforce and patient care

Journal:	*BMJ Open*
Manuscript ID	bmjopen-2017-021273
Article Type:	Protocol
Date Submitted by the Author:	20-Dec-2017
Complete List of Authors:	carrieri, daniele; Univeristy of Exeter Medical School<br>Briscoe, Simon; University of Exeter, University of Exeter Medical School<br>Jackson, Mark; University of Exeter College of Humanities, Wellcome Centre for Cultures and Environments of Health<br>Mattick, Karen; University of Exeter Medical School<br>Papoutsi, Chrysanthi; University of Oxford, Nuffield Department of Primary Care Health Sciences<br>Pearson, Mark; Hull York Medical School, Wolfson Palliative Care Research Centre<br>Wong, Geoffrey; Oxford, Nuffield Department of Primary Care Health SciencesUniversity of Oxford Radcliffe Primary Care Building Radcliffe Observatory Quarter
Keywords:	Health policy < HEALTH SERVICES ADMINISTRATION & MANAGEMENT, Protocols & guidelines < HEALTH SERVICES ADMINISTRATION & MANAGEMENT, MEDICAL EDUCATION & TRAINING

SCHOLARONE™
Manuscripts

'Care Under Pressure': a realist review of interventions to tackle doctors' mental ill-health and its impacts on the clinical workforce and patient care

Daniele Carrieri[1], Simon Briscoe[2], Mark Jackson[3], Karen Mattick[1], Chrysanthi Papoutsi[4], Mark Pearson[5], Geoff Wong[4]

1 University of Exeter Medical School, University of Exeter, Exeter, UK
2 Exeter HS&DR Evidence Synthesis Centre, Institute of Health Research, University of Exeter Medical School, Exeter, UK
3Wellcome Centre for Cultures and Environments of Health, University of Exeter, Exeter UK
4 Nuffield Department of Primary Care Health Sciences, University of Oxford, Oxford, UK
5 Wolfson Palliative Care Research Centre, Hull York Medical School, University of Hull, Hull, UK

Correspondence to: Daniele Carrieri; d.carrieri@exeter.ac.uk

Abstract

Introduction: Mental ill-health is prevalent across all groups of health professionals and this is of great concern in many countries. In the UK, the mental health of the National Health Service (NHS) workforce is a major healthcare issue, leading to presenteeism, absenteeism and loss of staff from the workforce. Most interventions targeting doctors aim to increase their 'productivity' and 'resilience', placing responsibility for good mental health with doctors themselves – and neglecting the organisational and structural contexts that may have a detrimental effect on doctors' wellbeing. There is a need for approaches that are sensitive to the contextual complexities of mental ill-health in doctors, and that do not treat doctors as a uniform body, but allow distinctions to account for particular characteristics, such as specialty, career stage, and different working environments.

Methods and analysis: Our project aims to understand how, why and in what contexts support interventions can be designed to minimise the incidence of doctors' mental ill-health. We will conduct a realist review – a form of theory-driven interpretative systematic review – of interventions, drawing on diverse literature sources. The review will iteratively progress through 5 steps: 1) locate existing theories; 2) search for evidence; 3) select articles; 4) extract and organise data; 5) synthesise evidence and draw conclusions. The analysis will summarise how, why and in what circumstances doctors' mental ill-health is likely to develop and what can remediate the situation. Throughout the project, we will also engage iteratively with diverse stakeholders in order to produce actionable theory.

Ethics and dissemination: Ethical approval is not required for our review. Our dissemination strategy will be participatory. Tailored outputs will be targeted to: policy makers; NHS employers and healthcare leaders; team leaders; support organisations; doctors experiencing mental ill-health, their families and colleagues.

For peer review only - http://bmjopen.bmj.com/site/about/guidelines.xhtml

Strengths and limitations of this study

- This is the first realist review of interventions to tackle the pressing problem of mental ill-health in doctors.
- Most published literature to date has tended to focus on workplace interventions aimed at increasing doctors' 'resilience', placing responsibility for good mental health with doctors themselves, and neglecting the organisational and structural contexts that may have a detrimental effect on doctors' wellbeing.
- A realist review approach accounts for the complexity and many dimensions (e.g. individual, organisational, socio-cultural) of the problem of mental ill-health in doctors, and for particular characteristics, such as specialty, career stage, different working environments.
- The engagement of different audiences (e.g. policy makers, doctors, healthcare leaders) in refining the programme theory will support the development of contextually-sensitive strategies to tackle mental ill-health in doctors.
- Only studies published in the English language will be included.

For peer review only

For peer review only - http://bmjopen.bmj.com/site/about/guidelines.xhtml

Introduction

'The most tragic thing in the world is a sick doctor'

G. B. Shaw 'The Doctor's Dilemma' (1).

A universal truth: no health without a *healthy* workforce

'A universal truth: no health without a workforce' is the compelling title of a 2013 World Health Organisation's (WHO) report on how the availability of healthcare staff underpins efforts to implement universal health coverage (2). For the purposes of our research, we wish to expand this 'universal truth' to argue that there can be no health without a *healthy* workforce.

Because of its centrality to the delivery of excellent, equitable, and increasingly complex healthcare (due also to biomedical innovation, ageing populations and the increase in multi-morbidity), the clinical workforce is a focus of interest both globally, and at the level of individual countries (3-5). However, like the abovementioned WHO report, most of this research is driven by quantitative measures such as supply and demand projections based on demographics, disease incidence, and the anticipated need for clinical workforce – but does not pay sufficient attention to an equally important factor: the clinical workforce's wellbeing (6).

Nevertheless, the wellbeing of the clinical workforce is becoming a major healthcare issue – and this is shown by the growing incidence of mental ill-health (e.g. stress, burnout, depression, drug and alcohol dependence, and suicide) across all groups of health professionals, and in many countries (7-11). A 2014 study conducted by the American Medical Association and Mayo Clinic researchers reported that 54% of physicians in the US

For peer review only - http://bmjopen.bmj.com/site/about/guidelines.xhtml

are experiencing professional burnout – a higher rate than other professions (12). Suicide rates are also high: a review of four decades of studies on physician's suicide estimated that the chances of dying by suicide are 70% higher than the general population for male physicians and 250-400% for female physicians (13).

The wellbeing of clinical workforce is not only an important issue in itself, but can significantly impact workforce projections, the cost of healthcare, and the quality of the care received by patients (6). The 'Triple Aim' of improving the health of the population, improving patient experience, and reducing cost, is a widely adopted guidance to optimise healthcare services' performance with rising patient needs, financial constraints, and workforce projections (14). Bodenheimer and Sinsky argued for the importance of adding to this triad the 'Fourth Aim' of *improving the work life of healthcare professionals* – noting how the positive engagement of the clinical workforce is key to achieve the health of the population (i.e. the 'First Aim') (15). Similar arguments underpin recent calls for internationally coordinated research efforts to develop evidence based strategies to tackle the high incidence of mental ill-health among healthcare professionals at a global level (16, 17).

In the UK the mental health of the National Health Service (NHS) workforce is of particular concern (18-22). In 2015 the Head of Thought Leadership at the King's Fund declared that stress levels among NHS staff are "astonishingly high " and require to be treated as a "public health problem" (23). In a similar vein to the international literature sketched above, the recent 2017 Lord Select Committee's report on the sustainability of the NHS and Adult Social care (24) states that "the absence of any comprehensive national long-term strategy

For peer review only - http://bmjopen.bmj.com/site/about/guidelines.xhtml

to secure the appropriately skilled, well-trained and committed workforce […] represents the biggest internal threat to the sustainability of the NHS" (p.35).

When faced with mental ill-health, healthcare professionals may feel they have to continue caring for patients despite their own difficulties (presenteeism)(25-27), or they may have to take sickness leave, which could result in gaps in the service (absenteeism)(28), or leave the NHS either temporarily or permanently (workforce retention)(29-32). Although mental ill-health is prevalent amongst all groups of healthcare professionals working in the NHS, our research focuses on doctors across specialties and career stages. This focus reflects the current recruitment and workforce retention issues (e.g. doctors-in-training, general practice, emergency medicine), the significant potential for sick doctors to inadvertently cause harm to patients, and the financial implications of doctors' mental ill-health (8, 21).

Why are doctors particularly at risk of mental ill-health?

Peer-reviewed and grey literature highlights a large number of individual, occupational, and broader causative risk factors leading to mental ill-health which operate at a socio-cultural level (29, 33, 34).

Overall, such factors include: the emotionally demanding nature of the profession (28, 35); the increasing workload resulting from attempting to provide more, and higher quality, care on shrinking budgets (36); systems of clinical governance which are leading to loss of autonomy and erosion of professional values (37); rigid organisational structures and inflexible working hours (38) and; highly bureaucratic professional regulatory systems (e.g. appraisals, revalidation, quality inspection visits etc.) (39). Doctors are also at higher risk than the general population to develop addiction and substance misuses because of their knowledge of and access to drugs, and potential to self-medicate (18). All these factors may be intensified by a doctors' tendency to avoid seeking help and support when unwell or

For peer review only - http://bmjopen.bmj.com/site/about/guidelines.xhtml

under pressure (40, 41), and by a perceived stigma amongst doctors around mental illness (10, 42).

The factors associated with mental ill-health and decisions to leave the medical profession have been described as heavy workload, long working hours, high levels of regulation and scrutiny, perceived reduced autonomy and fear of complaints and negligence claims (29, 30). Presenteeism in doctors may be underpinned by a fear of career repercussions, a fear of letting down colleagues and patients, the difficulties of arranging cover, a failure to prioritise their own health needs and a failure to recognise their own vulnerability to illness (22, 43). It seems that doctors may feel pressurised by collective norms to be present but it is currently unclear whether this varies at different career stages or in different specialties.

Current interventions and gaps

There is a large literature on interventions that offer support, advice and/or treatment to doctors living with mental health difficulties, and that addresses the associated impacts such as presenteeism, absenteeism and workforce retention (31, 34). Most of this literature tends to focus on workplace interventions aimed at increasing doctors' 'productivity' and 'resilience', placing responsibility for good mental health with doctors themselves (21, 44, 45). Such a tendency – which mirrors broader socio-political strategies and discourses (46, 47) – neglects the organisational and structural contexts that may have a detrimental effect on doctors' wellbeing. This can potentially aggravate work-related pressure, leading to mental-ill-health.

Some scholars suggest that interventions should focus on organisational support and systemic factors contributing to mental ill-health, rather than on individual doctors (22) – highlighting the need to think in terms of 'organisational resilience' (45).

From a systems level, Wallace et al. (11) categorise interventions into workplace and

For peer review only - http://bmjopen.bmj.com/site/about/guidelines.xhtml

profession awareness, management, and prevention; physician self-care and prevention; physician treatment and recovery; and improved patient care and system outcomes.

As the ‘culture of medicine’ starts early in undergraduate medical programmes, doctors-in-training are affected both directly (e.g. by becoming ill themselves) and indirectly (e.g. through their colleagues being ill) by mental ill-health. Therefore strategies should also start early in a doctor’s career, with medical training emphasising pathways for help and increasing awareness – and de-stigmatisation – of mental illness in doctors (48).

This knowledge of interventions that offer support, advice and/or treatment to doctors experiencing mental ill-health has not been synthesised in a way that takes account of their complexity and heterogeneity. Currently, it is not clear which components within these interventions matter more (or less) than others, for whom they matter and in what contexts. For example, a given intervention might work well for some doctors and not others (which might be influenced by personal factors such as age, gender, seniority); and in some contexts and not others (as it might be influenced by organisational factors such as the degree of organisational change or societal factors such as recent media portrayal).

Therefore there is a need for research approaches that are sensitive to the contextual complexities of the problem of mental ill-health in doctors. These methodologies should not treat doctors as a uniform body, but they should allow distinctions to account for particular characteristics, such as specialty and career stage, and different working environments.

Methods and Analysis

Project Aim

This research aims to improve understanding of how, why and in what contexts mental

For peer review only - http://bmjopen.bmj.com/site/about/guidelines.xhtml

health services and support interventions can be designed in order to minimise the incidence of doctors' mental ill-health.

Project Objectives

1. To conduct a realist review on interventions to tackle doctors' mental ill-health and its impacts on the clinical workforce and patient care, drawing on diverse literature sources and engaging iteratively with diverse stakeholder perspectives to produce actionable theory.
2. To produce recommendations that support the tailoring, implementation, monitoring and evaluation of contextually-sensitive strategies to tackle mental ill-health and its impacts.

Review questions

1. What are the processes by which mental ill-health in doctors develops and leads to its negative impacts, and where are the gaps that interventions do not address currently?
2. What are the mechanisms, acting at individual, group, profession, and organisational levels, by which interventions to reduce doctors' mental ill-health at the different stages are believed to result in their intended outcomes?
3. What are the important contexts which determine whether the different mechanisms produce the intended outcomes?
4. What changes are needed to existing and/or future interventions to make them more effective?

For peer review only - http://bmjopen.bmj.com/site/about/guidelines.xhtml

Research Plans

<u>Objective 1. To conduct a synthesis of the literature using a realist review approach</u>

Any evidence synthesis that seeks to make sense of interventions aiming to improve doctors' mental ill-health must take into account the contexts in which these interventions are situated. This will generate an in-depth understanding of which components within these interventions matter more (or less) than others, for whom they matter and in what ways. A realist review can synthesize relevant data found within qualitative, quantitative and mixed-methods research. By following an interpretive, theory-driven approach to analysing data from such diverse literature sources, realist reviews move beyond description, to provide findings that coherently and transferably explain *how* and *why* contexts can influence outcomes.

The plan of investigation will follow a detailed realist review protocol informed by Pawson's five iterative stages in realist reviews (49) and the RAMESES quality and publication standards for realist reviews (50, 51).

The realist review protocol is registered with PROSPERO (52). The review process also incorporates iterative cycles of engagement with the literature and with our Stakeholder Group (comprising clinicians, service users, senior NHS managers, therapists working with 'sick doctors', policy makers and charities), who will provide their own perspectives on the positive and negative interactions between healthcare contexts, the development of mental ill-health in doctors, and the subsequent impacts such as presenteeism, absenteeism and workforce retention. These cycles of engagement will enable the production of action-oriented middle-range theory which can inform change at individual, group, profession, and organisational levels (see also Objective 2 below).

For peer review only - http://bmjopen.bmj.com/site/about/guidelines.xhtml

Step 1: Locate existing theories

The goal of this step is to identify theories that explain how interventions aiming to support doctors challenged by mental ill-health are supposed to work (and for whom), when they do work, when they do not achieve the desired change in practice, why they are not effective, and why they are not being used. The rationale for this step is that interventions are "theories incarnate" – that is, such interventions are underpinned by assumptions about why certain components are required. In other words, the designers of interventions have put them together in a certain way based on their theories about what needs to be done to get one or more desired outcomes (53).

To locate these theories, in the first instance we will iteratively: a) draw on ongoing qualitative interviews (already conducted by DC) with the clinical team of therapists working at the NHS Practitioner Health Programme[1] ; b) consult with key content experts representing multidisciplinary perspectives in our Stakeholder Group and; c) draw on an exploratory search of relevant literature.

Building the programme theory will require iterative discussions within the project team to make sense of and synthesise the different theories into an initial programme theory. The project team will also organise stakeholder and 'sense-making' meetings to discuss and refine the programme theory.

Step 2: Search for evidence

[1] A national centre that provides support to doctors and other healthcare professionals experiencing mental ill-health (for more information visit http://php.nhs.uk/)

For peer review only - http://bmjopen.bmj.com/site/about/guidelines.xhtml

Formal search

The purpose of this Step is to find a relevant 'body of literature' that might contain data with which to further develop and refine the programme theory from Step 1. Searching will be designed, piloted and conducted by an information specialist (SB).

We anticipate that we may need to search the following databases: MEDLINE, MEDLINE-in-Process, PsycINFO, ASSIA and any other relevant databases identified by the information specialist. We will also undertake forward citation searches and search the citations contained in the reference lists of relevant documents. We anticipate that we will search the databases using search terms for "doctors", "mental ill-health", "absenteeism", "presenteeism" and "workforce retention", although the exact terminology, syntax and search structure will be determined by the results of Step 1. Subject headings relevant to each database will also be used, for example, MeSH for MEDLINE.

Screening

We will include literature relating to all doctors from the outset. We believe greater explanatory insight might be attained by looking across stages of training and across specialties, particularly since our preliminary work suggests common mechanisms may be at play in different settings (e.g. inflexible working patterns, wider NHS culture).

The following initial inclusion criteria will be applied:

- Mental ill-health and its impacts (e.g. presenteeism, absenteeism, workforce retention) –all studies that focused on one or more of these aspects. Note, generic occupational health services targeting whole populations of doctors, rather than doctors experiencing mental ill-health for doctors, would not be included.
- Study design – all study designs.
- Types of settings – all healthcare settings.

For peer review only - http://bmjopen.bmj.com/site/about/guidelines.xhtml

- Types of participants – all studies that included medical doctors.
- Types of intervention – interventions or resources that focus on improving mental ill-health and minimizing its impacts.
- Outcome measures – all mental health outcomes and measures relevant to its impacts (e.g. absenteeism, presenteeism, workforce retention).

Screening will be undertaken by DC. A 10% random sub-sample of the citations retrieved from searching will be reviewed independently for consistency by CP. Any disagreements will be resolved by discussion between the DC and CP (the second reviewer). If disagreements remain then the matter will be presented to the whole project team for discussion and resolved by majority vote.

Additional searching

An important process in realist reviews is finding additional data to confirm, refine or refute aspects of developing programme theory. More searches will be undertaken if we find that we require more data to develop and confirm, refute or refine certain sub-sections of the programme theory. To learn more about the influence of wider contexts on mental ill-health and its impacts, we may also look at literature about doctors working in other countries, other groups of healthcare professionals working in the UK and professions outside healthcare who experience the same broader societal changes but in a different industry. Searches may also seek to identify 'good practice' examples in healthcare, where mental ill-health of some institutions is particularly low (e.g.(54)). For each additional search the project team will meet to discuss and set inclusion and exclusion criteria. Different search terms and databases are likely to be needed for these purposive searches which will be developed, piloted and conducted in conjunction with our information specialist (SB). The

For peer review only - http://bmjopen.bmj.com/site/about/guidelines.xhtml

screening processes will be as described above. Where applicable, we will follow the search strategies proposed by Booth et al. which have been developed for such data (55).

Step 3: Article selection

Documents will be selected based on relevance (whether data can contribute to theory building and/or testing) and rigour (whether the methods used to generate the relevant data are credible and trustworthy)(53). Even when a document from the initial search has been screened and has met inclusion criteria, it may still not contain any data that is relevant for programme theory development and refinement.

Included papers would be divided into those which can make ‘major’ or ‘minor’ contributions to our research question. For example, we may classify as ‘major’ those studies conducted in countries where doctors predominantly work in universal, publicly-funded health care systems with similarities to the NHS; or those where the mechanisms (which cause doctors’ mental ill-health to develop) are similar, even if they are operating in different contexts. This will enable us to focus effort on the studies which make a major contribution, whilst ensuring that we do not miss any important relevant data from the wider literature. In this way we will inevitably prioritise studies from the UK but also include studies from other countries that provide useful insights for the UK. This strategy will enable us to be rigorous while keeping the project manageable. Our *provisional* criteria for classifying studies as ‘major’ or ‘minor’ are:

Major:

- Studies which contribute to the research questions and are conducted in an NHS context.

For peer review only - http://bmjopen.bmj.com/site/about/guidelines.xhtml

- Studies which contribute to the research questions and are conducted in contexts (e.g. universal, publicly-funded health-care systems) with similarities to the NHS.
- Studies which contribute to the research questions and can clearly help to identify mechanisms which could plausibly operate in the context of the NHS.

Minor:

- Studies conducted in health-care systems that are markedly different to the NHS (e.g. fee-for service, private insurance scheme systems) but where the mechanisms could plausibly operate in the context of doctors working in the NHS.

Classification decisions will be checked between two reviewers (DC and CP) and discussed with the rest of the team. A random sample of 10% of documents will be selected, assessed and discussed between the DC and CP to ensure that decisions for final inclusion have been made consistently. The remaining 90% of decisions will be made by DC. We will employ the same decision making process as outlined above in Step 2. Article selection for any additional searches will follow the process described above.

Step 4: Extracting and organising data

The full texts of the included papers will be uploaded in NVivo QRS International (a qualitative data management software). Relevant sections of texts interpreted as contexts, mechanisms and/or their relationships to outcomes will be coded in NVivo. At the initial stages the coding will be both inductive (codes created to categorise data reported in included studies) and deductive (codes created in advance of data extraction and analysis as informed by the initial programme theory)(56).The main analysis of the realist review will be retroductive. Each new element of relevant data will be used to refine

For peer review only - http://bmjopen.bmj.com/site/about/guidelines.xhtml

aspects of the programme theory, and as it is refined, included studies and documents will (where necessary) be re-scrutinised to search for data relevant to the revised programme theory that may have been missed initially. The characteristics of the studies and interventions will be extracted separately into an Excel spreadsheet to provide a descriptive overview.

We shall also extract (from included documents) all data on the cost of various interventions to tackle doctors' mental ill-health, but we shall not undertake a formal health economic assessment. Our goal is to identify what data exist on costs and also if any of these are useful in helping us to suggest any implications for policy and practice. During the review process, we will extract the following types of economic data or information (where available):

- Direct costs of interventions;
- Indirect costs relating to the intended beneficial effects of interventions (accessing mental health services, Occupational Health consultations, and so on)
- Unit costs and total costs
- Currency
- Time period to which economic data relates.

The way that economic evaluations are conducted and reported makes it unlikely to be possible to link the data in any included economic evaluations directly to the context - mechanism-outcome configurations identified in the realist review. We therefore anticipate presenting the cost information, where it is available, separately from the CMO configurations, which will make it easily accessible to readers interested in this particular area.

For peer review only - http://bmjopen.bmj.com/site/about/guidelines.xhtml

For peer review only

DC will undertake data extraction and organisation. A random sample of 10% of coded documents will be independently checked by CP for consistency. Any disagreements will be resolved by discussion between the DC and CP. If disagreements still remain then a third member of the project team will be asked for their opinion and resolution will be by majority vote. We will start the coding and analysis process by using the literature that has been deemed to make a 'major' contribution to the research questions to continue building and refining our programme theory, while progressively focusing the review. Articles categorised as providing 'minor' contributions will be analysed to address particular aspects of the programme theory where necessary. The aim of the analysis will be to reach theoretical saturation in understanding the problem of mental ill-health, rather than to aggregate every single study that exists in the area. Decisions about whether a study can have a 'major' or 'minor' contribution may change over the course of the project, as the analysis progresses. All changes will be documented and recorded as part of an audit trail to increase transparency and ensure consistency.

Step 5: Synthesising the evidence and drawing conclusions

In Step 5 we will continue to use a realist logic of analysis to build context-mechanism-outcome configurations (CMOCs). These will aim to explain the outcomes resulting from the intervention strategies discussed in the included documents. For example, we will use interpretative cross-case comparison to explain how and why observed outcomes have occurred, by comparing interventions where reducing mental ill-health has been 'succesful' against those which have not, to understand how context has influenced reported findings. To achieve this, we will continue to interpret the data to ascertain if it pertains to contexts (C), mechanisms (M), outcomes (O), the relationships between C, M, and O and/or the

For peer review only - http://bmjopen.bmj.com/site/about/guidelines.xhtml

relationships between CMOCs (53). This type of analysis will enable us to understand the behaviour of the most relevant and important mechanisms under different contexts, thus allowing us to build more transferable CMOCs. We will be drawing on substantive and formal theory to inform programme theory development.

During the review, we move iteratively between the analysis of particular examples from the literature, refinement of programme theory, and further iterative searching for data to test particular subsections of the programme theory.

Finally, when making sense of our data during analysis we will use the following analytic thinking processes (57):

a) Juxtaposition of sources of evidence – for example, where evidence about behaviour change in one source enables insights into evidence about outcomes in another source.

b) Reconciling of sources of evidence – where results differ in apparently similar circumstances, further investigation is appropriate in order to find explanations for why these different results occurred.

c) Adjudication of sources of evidence – on the basis of methodological strengths or weaknesses.

d) Consolidation of sources of evidence – where outcomes differ in particular contexts, an explanation can be constructed of how and why these outcomes occur differently.

For peer review only - http://bmjopen.bmj.com/site/about/guidelines.xhtml

Objective 2: Design contextually-sensitive strategies to tackle mental ill-health and its impacts on doctors, their colleagues and their patients.

We will use the 'Evidence Integration Triangle' (EIT) (58) as a framework for bringing together stakeholders around evidence in a collaborative, action-oriented way. Using the EIT will enable us to create a facilitative environment in which research can inform practical decision-making, and for experiential knowledge from lived experiences and from professional practice to inform interpretation of that research.

We will use the three components of the EIT (1. practical evidence-based interventions; 2. pragmatic, longitudinal measures of progress; and 3. participatory implementation processes) to structure and inform the facilitation of the Stakeholder Group meetings and a workshop with policy makers. The timing of these meetings has been selected to maximise input to the realist review process and enable local, regional and national dissemination at the most appropriate stages of the project.

Dissemination

We would like to engage different types of audiences and the key messages and communication strategies will be tailored to respond to their needs. The Stakeholder Group will be well placed to advise on the key audiences and how we should target messages to that audience. We will also draw on existing networks and communication strategies, for example existing links with clinicians and professional bodies, wherever possible to reach the widest possible number of beneficiaries. So far we have identified 5 key audiences that we would like to engage and reach with our dissemination strategy:

For peer review only - http://bmjopen.bmj.com/site/about/guidelines.xhtml

- Group 1: Policy makers who can influence change that will affect doctors at a national level.
- Group 2: Employers and healthcare leaders who can shape the structure of organisations in which doctors work.
- Group 3: Team leaders who can shape the immediate working environment for individual doctors.
- Group 4: National, regional and local groups and organisations that provide support to doctors experiencing mental ill-health.
- Group 5: Doctors who are experiencing mental ill-health, and their families and colleagues.

We want to ensure that this project's outputs will be useful to the NHS and will address this by producing outputs that are deemed appropriate and relevant by our different groups of stakeholders, and acknowledging likely implementation barriers. The project will produce five major types of output. We will consult with our Stakeholder Group and use their knowledge and experience to refine the development, presentation and dissemination of these outputs:

1. Conventional academic forms. A report for publication in the NIHR HS&DR Journal; a report for publication in a high-impact peer-reviewed journal; and conference presentations. This will inform the agenda for debate and action in health services and in public policy more widely (Groups 1-5).
2. More innovative forms. Depending on the results of the realist review, we propose to translate some of our outputs into comics, animations and/or information graphics that might be distributed more widely (e.g. for notice boards on wards,

For peer review only - http://bmjopen.bmj.com/site/about/guidelines.xhtml

inductions, teaching sessions) to raise awareness and normalise mental health issues. As demonstrated previously, comics can provide an appropriate format for tackling delicate issues such as mental ill-health (59) (Group 5).

3. Measures/indicators. This builds on our use of the Evidence Integration Triangle to inform our interpretation/dissemination strategy and would be offered for use in existing systems to monitor and evaluate the impact of changes made based on our research findings. This will enable frontline staff and managers to implement and monitor the impact of research-informed changes in practice (Groups 1-4).
4. Plain English summaries. The research findings would be tailored to different audiences (e.g. doctors, patients, health service managers, medical educators, policy makers). This will provide a meaningful summary of findings which increase stakeholders' recognition and understanding of the issue and how evidence can inform actions they can take (Groups 1-5).

Acknowledgments: This project was funded by the National Institute for Health research Service and Delivery Research (project number 16/53/12).

Authors Contribution: KM and MP conceptualised the study with input from SB, CP, DC, MJ, and GW. DC wrote the first draft of this manuscript. SB, CP, KM, MP, and GW critically contributed to and refined this manuscript. SB, CP, DC, KM, MP, MJ, and GW have read and approved the final manuscript.

Funding: Health Service and Delivery Research (HS&DR) Programme

Competing Interests: None declared

Disclaimer: The views and opinions expressed therein are those of the authors and do not necessarily reflect those of the NH&DR Programme, NIHR, NHS or the Department of Health.

Ethics approval: The project has been reviewed by the relevant University of Exeter Ethics Committee

Prospero registration number CRD42017069870

For peer review only - http://bmjopen.bmj.com/site/about/guidelines.xhtml

References

1. Shaw B. The doctor's dilemma: Brentano's; 1911.
2. Campbell J, Dussault G, Buchan J, Pozo-Martin F, Guerra Arias M, Leone C, et al. A universal truth: no health without a workforce. Forum report, Third Global Forum on Human Resources for Health, Recife, Brazil. Global Health Workforce Alliance and World Health Organization, Geneva. 2013.
3. World Health Organisation (WHO) National Health Workforce Accounts: A Handbook. http://www.who.int/hrh/documents/brief_nhwfa_handbook/en/; 2016.
4. World Health Organisation (WHO) [internet]. http://www.who.int/hrh/statistics/en/: [cited 12/12/2017]; 2016.
5. Chambers C, Frampton C, Barclay M. Presenteeism in the New Zealand senior medical workforce-a mixed-methods analysis. The New Zealand medical journal. 2017;130(1449):10-21.
6. Kreitzer MJ, Klatt M. Educational innovations to foster resilience in the health professions. Medical Teacher. 2017;39(2):153-9.
7. Haramati A, Cotton S, Padmore JS, Wald HS, Weissinger PA. Strategies to promote resilience, empathy and well-being in the health professions: Insights from the 2015 CENTILE Conference. Medical teacher. 2017;39(2):118-9.
8. Anonymous (editorial) The future of the NHS. The Lancet. 2017.
9. Shanafelt TD, Hasan O, Dyrbye LN, Sinsky C, Satele D, Sloan J, et al. Changes in Burnout and Satisfaction With Work-Life Balance in Physicians and the General US Working Population Between 2011 and 2014. Mayo Clinic proceedings. 2015;90(12):1600-13.
10. Hayes B, Prihodova L, Walsh G, Doyle F, Doherty S. What's up doc? A national cross-sectional study of psychological wellbeing of hospital doctors in Ireland. BMJ open. 2017;7(10):e018023.
11. Wallace JE, Lemaire JB, Ghali WA. Physician wellness: a missing quality indicator. The Lancet. 2009;374(9702):1714-21.
12. Shanafelt TD, Gorringe G, Menaker R, Storz KA, Reeves D, Buskirk SJ, et al. Impact of organizational leadership on physician burnout and satisfaction. Mayo Clin Proc. 2015;90(4):432-40.
13. Hampton T. Experts address risk of physician suicide. Jama. 2005;294(10):1189-91.
14. Berwick DM, Nolan TW, Whittington J. The triple aim: care, health, and cost. Health Aff (Millwood). 2008;27(3):759-69.
15. Bodenheimer T, Sinsky C. From triple to quadruple aim: care of the patient requires care of the provider. Annals of family medicine. 2014;12(6):573-6.
16. Dyrbye LN, Trockel M, Frank E, et al. Development of a research agenda to identify evidence-based strategies to improve physician wellness and reduce burnout. Annals of Internal Medicine. 2017;166(10):743-4.
17. Lemaire JB, Wallace JE. Burnout among doctors. Bmj. 2017;358:j3360.
18. Marshall EJ. Doctors' health and fitness to practise: treating addicted doctors. Occupational Medicine. 2008;58(5):334-40.
19. Brooks SK, Gerada C, Chalder T. The specific needs of doctors with mental health problems: qualitative analysis of doctor-patients' experiences with the Practitioner Health Programme. Journal of Mental Health. 2017;26(2):161-6.

For peer review only

For peer review only - http://bmjopen.bmj.com/site/about/guidelines.xhtml

20. Braquehais MD, Eiroa-Orosa FJ, Holmes KM, Lusilla P, Bravo M, Mozo X, et al. Differences in Physicians' and Nurses' Recent Suicide Attempts: An Exploratory Study. Archives of Suicide Research. 2016;20(2):273-9.
21. Wilkinson E. UK NHS staff: stressed, exhausted, burnt out. The Lancet. 2015;385(9971):841-2.
22. Spiers J, Buszewicz M, Chew-Graham CA, Gerada C, Kessler D, Leggett N, et al. Barriers, facilitators, and survival strategies for GPs seeking treatment for distress: a qualitative study. Br J Gen Pract. 2017;67(663):e700-e8.
23. Limb M. Stress levels of NHS staff are "astonishingly high" and need treating as a public health problem, says King's Fund. BMJ Careers. 2015;Available at: http://careers.bmj.com/careers/advice/Stress_levels_of_NHS_staff_are_%E2%80%9Castonishingly_high%E2%80%9D_and_need_treating_as_a_public_health_problem,_says_King%E2%80%99s_Fund
24. House of Lords, The Long-term Sustainability of the NHS and Adult Social Care. 2017.
25. Oxtoby K. Why doctors don't take sick leave. BMJ. 2015;351.
26. Oxtoby K. Why doctors need to resist" presenteeism". BMJ: British Medical Journal (Online). 2015;351.
27. Davies M. Medicolegal aspects of working while unwell. BMJ: British Medical Journal (Online). 2015;351.
28. Weinberg A, Creed F. Stress and psychiatric disorder in healthcare professionals and hospital staff. the Lancet. 2000;355(9203):533-7.
29. Doran N, Fox F, Rodham K, Taylor G, Harris M. Lost to the NHS: a mixed methods study of why GPs leave practice early in England. Br J Gen Pract. 2016;66(643):e128-e35.
30. Smith F, Lachish S, Goldacre MJ, Lambert TW. Factors influencing the decisions of senior UK doctors to retire or remain in medicine: national surveys of the UK-trained medical graduates of 1974 and 1977. BMJ open. 2017;7(9):e017650.
31. Silver MP, Hamilton AD, Biswas A, Warrick NI. A systematic review of physician retirement planning. Human resources for health. 2016;14(1):67.
32. Lambert TW, Smith F, Goldacre MJ. Why doctors consider leaving UK medicine: qualitative analysis of comments from questionnaire surveys three years after graduation. Journal of the Royal Society of Medicine. 2017:141076817738502.
33. Brooks SK, Gerada C, Chalder T. Review of literature on the mental health of doctors: are specialist services needed? Journal of mental health. 2011;20(2):146-56.
34. Rothenberger DA. Physician Burnout and Well-Being: A Systematic Review and Framework for Action. Diseases of the colon and rectum. 2017;60(6):567-76.
35. Riley R, Weiss MC. A qualitative thematic review: emotional labour in healthcare settings. Journal of advanced nursing. 2016;72(1):6-17.
36. Jones MC, Wells M, Gao C, Cassidy B, Davie J. Work stress and well-being in oncology settings: a multidisciplinary study of health care professionals. Psycho-oncology. 2013;22(1):46-53.
37. Freidson E. Doctoring together: A study of professional social control: Elsevier; 1975.
38. Leape L, Berwick D, Clancy C, Conway J, Gluck P, Guest J, et al. Transforming healthcare: a safety imperative. Quality and Safety in Health Care. 2009;18(6):424-8.
39. Department of Health. Mental health and ill health in doctors. London, UK; 2008.
40. Garelick AI. Doctors' health: stigma and the professional discomfort in seeking help. RCP; 2012.

For peer review only - http://bmjopen.bmj.com/site/about/guidelines.xhtml

41. Kay M, Mitchell G, Clavarino A, Doust J. Doctors as patients: a systematic review of doctors' health access and the barriers they experience. Br J Gen Pract. 2008;58(552):501-8.
42. Stanton J, Randal P. Developing a psychiatrist–patient relationship when both people are doctors: a qualitative study. BMJ open. 2016;6(5):e010216.
43. McKevitt C, Morgan M, Dundas R, Holland W. Sickness absence and working through illness: A comparison of two professional groups. Occupational Health and Industrial Medicine. 1998;1(38):20.
44. Balme E, Gerada C, Page L. Doctors need to be supported, not trained in resilience. BMJ Careers. 2015;15.
45. Cheshire A, Ridge D, Hughes J, Peters D, Panagioti M, Simon C, et al. Influences on GP coping and resilience: a qualitative study in primary care. Br J Gen Pract. 2017:bjgp17X690893.
46. Petersen A, Lupton D. The New Public Health. Discourses, Knowledges, Strategies: Sage; 1997.
47. Jackson M. The age of stress: science and the search for stability: Oxford University Press; 2013.
48. Meyers MF. Why physicians die by suicide: lessons learned from their families and others who cared: Myers Publisher; 2017.
49. Pawson R, Greenhalgh T, Harvey G, Walshe K. Realist synthesis: an introduction. Manchester: ESRC Research Methods Programme, University of Manchester. 2004.
50. Wong G, Greenhalgh T, Westhorp G, Pawson R. Development of methodological guidance, publication standards and training materials for realist and meta-narrative reviews: the RAMESES (Realist And Meta-narrative Evidence Syntheses–Evolving Standards) project. 2014.
51. Wong G, Westhorp G, Manzano A, Greenhalgh J, Jagosh J, Greenhalgh T. RAMESES II reporting standards for realist evaluations. BMC Medicine. 2016;14(1):96.
52. Booth A, Clarke M, Ghersi D, Moher D, Petticrew M, Stewart L. An international registry of systematic-review protocols. The Lancet. 2011;377(9760):108-9.
53. Pawson R. Evidence-based policy: a realist perspective: Sage; 2006.
54. Monrouxe LV, Rees CE. Healthcare professionalism: improving practice through reflections on workplace dilemmas: John Wiley & Sons; 2017.
55. Booth A, Croot E, Wilkins E, Campbell F, Springett J, Harris J. Towards a methodology for cluster searching to provide conceptual and contextual “richness” for systematic reviews of complex interventions: case study (CLUSTER). BMC medical research methodology. 2013;13(1):118.
56. Braun V, Clarke V. Using Thematic Analysis in Psychology. Qualitative Research in Psychology. 2006;3:77-101.
57. Pawson R. The science of evaluation: a realist manifesto: Sage; 2013.
58. Glasgow RE, Green LW, Taylor MV, Stange KC. An evidence integration triangle for aligning science with policy and practice. American journal of preventive medicine. 2012;42(6):646-54.
59. Green MJ. Comics and medicine: peering into the process of professional identity formation. Academic Medicine. 2015;90(6):774-9.

For peer review only

For peer review only - http://bmjopen.bmj.com/site/about/guidelines.xhtml
