# Supplementary material for: ‘Care Under Pressure’: a realist review of interventions to tackle doctors’ mental ill-health and its impacts on the clinical workforce and patient care
Source: BMJ Open. 2018 Feb 2;8(2):e021273. doi: 10.1136/bmjopen-2017-021273 (PMC5829880; doi:10.1136/bmjopen-2017-021273)
Supplement: Reviewer comments [file bmjopen-2017-021273.reviewer_comments.pdf]

PEER REVIEW HISTORY

BMJ Open publishes all reviews undertaken for accepted manuscripts. Reviewers are asked to complete a checklist review form (**http://bmjopen.bmj.com/site/about/resources/checklist.pdf**) and are provided with free text boxes to elaborate on their assessment. These free text comments are reproduced below.

ARTICLE DETAILS

**TITLE (PROVISIONAL)**	'Care Under Pressure': a realist review of interventions to tackle doctors' mental ill-health and its impacts on the clinical workforce and patient care
**AUTHORS**	carrieri, daniele; Briscoe, Simon; Jackson, Mark; Mattick, Karen; Papoutsi, Chrysanthi; Pearson, Mark; Wong, Geoffrey

This article was not externally reviewed at BMJ Open. Protocols that have been independently assessed prior to submission to BMJ Open are usually fast-tracked to publication on the grounds that further substantial changes will not be possible. This independent assessment will usually be external, independent review for both a substantial grant award from a non-commercial or government funder and ethics approval.
